# Expression and prognostic significance of Golgiglycoprotein73 (GP73) with Epithelial-mesenchymal transition (EMT) related molecules in Hepatocellular Carcinoma (HCC)

**DOI:** 10.1186/1746-1596-8-197

**Published:** 2013-12-06

**Authors:** Yong xing Bao, Qian Cao, Ying Yang, Rui Mao, Lei Xiao, Hua Zhang, Hua-rong Zhao, Hao Wen

**Affiliations:** 1 Tumor Department, First Affiliated Hospital of Xinjiang Medical University (XJMU), Urumqi, China

## Abstract

**Background:**

Hepatocellular carcinoma (HCC) is the fifth most common cancer and the third cause of cancer-related deaths, worldwide. It is essential to develop an effective prognostic biomarker and determine the mechanisms underlying HCC invasion and metastasis.

**Aims:**

This study aimed to investigate the expression of Golgi glycoprotein73 (GP73) and Epithelial-mesenchymal transition (EMT) molecules such as E-cadherin and Vimentin in HCC. We also evaluated the prognostic value of GP73 in HCC.

**Methods:**

Immunohistochemistry (IHC) was used to determine the expression of GP73 and EMT molecules in 75 HCC specimens and the corresponding paracarcinomatous liver (PCL) tissues. Spearman’s correlation test was used to analyze the correlation of GP73 and EMT molecules. Clinicopathological features of the HCC patients were also analyzed. Univariate survival analysis was performed by the Kaplan–Meier method and differences among the groups were analyzed by the Log-rank test.

**Results:**

GP73 expression in HCC was higher compared with PCL tissues (*χ*^*2*^ = 73.60, *P* < 0.05). EMT molecules were also detected in HCC and PCL tissues. GP73 was negatively correlated with E-cadherin (*r* = − 0.49, *P* < 0.05), but positively correlated with Vimentin (*r* = 0.46, *P* < 0.05) in HCC. GP73 was correlated with the clinicopathological features including Edmondson grade, vascular invasion and TNM stage (P < 0.05), which was also associated with overall survival (OS) (*P* < 0.05).

**Conclusions:**

GP73 was negatively with E-cadherin and positively correlated with Vimentin. It might be associated with aggressive behavior of HCC and had influence on patients’ OS. Further research is needed to determine the potential of GP73.

**Virtual slides:**

The virtual slide(s) for this article can be found here: http://www.diagnosticpathology.diagnomx.eu/29 vs/1504046946108618; http://med.motic.com/MoticGallery/Slide?id=3b6a037e-f60e-4c68-9106-41e790de9431&user=2C69F0D6-A478-4A2B-ABF0-BB36763E8025; http://med.motic.com/MoticGallery/Slide?id=a25b5b32-b613-47b0-9f8b-e1e67a95d1bf&user=2C69F0D6-A478-4A2B-ABF0-BB36763E8025.

## Background

Hepatocellular carcinoma (HCC) accounts for most liver cancers worldwide, with poor prognosis [1]. The poor prognosis is attributed to extensive regional invasion and distant metastasis during the initial diagnosis. However, the mechanism underlying local invasion and distant metastasis are still unclear. Therefore, the need for effective molecular markers to evaluate the prognosis of HCC cannot be overstated.

Recently, a new biomarker Golgi glycoprotein73 (GP73) has been investigated for its diagnostic accuracy and potential clinical application in HCC. GP73 was first discovered in 2000 [2]. Many reports suggest that the GP73 expression was significantly increased in HCC unlike normal human liver cells [3]. The sensitivity and specificity of GP73 were higher than AFP, making it an ideal marker for early diagnosis of HCC [2,4,5]. However, research studies of GP73 until now have focused merely on its role in early diagnosis. Reports investigating the role of GP73 in clinical pathology and patients’ OS are rare.

Epithelial–mesenchymal transition (EMT) is a useful prognostic marker for survival in patients. EMT has a close relationship with tumor invasiveness and metastasis. The most representative molecules are E-cadherin and Vimentin [6-8].

Therefore, we explored the expression of GP73, E-cadherin and Vimentin in HCC /PCL tissues. The aim of the study was to find the relationships among GP73 and EMT molecules and to evaluate the role of GP73 in predicting the prognosis in HCC.

## Methods

### Patients and tissue samples

A total of 75 samples, including HCC and PCL tissues were obtained by surgical resection from the First Affiliated Hospital of Xinjiang Medical University (XJMU), between 2007 and 2012. Insufficient liver tissue in the biopsy specimen or insufficient clinical data regarding patient outcomes, were exclusion criteria. None of the patients received chemotherapy or radiotherapy prior to surgery. All patients were followed-up via telephone or questionnaires. Survival was calculated from tissue diagnosis until the patient’s death or termination of follow-up (April 2013). We obtained consent from all patients, and determined the clinic pathology including gender, age, AFP, HBV infection, thrombosis, tumor differentiation, vascular invasion and TNM stage. The TNM classification system was in accordance with the American Joint Committee on Cancer/International Union Against Cancer (AJCC/UICC). The tumor differentiation was based on the Edmondson grading.

The tissue specimens were resected from the HCC patients. We obtained the consent of all patients. Research carried out on patients was in compliance with the Helsinki Declaration and approved by the Xinjiang Medical Ethics Committee.

### Reagents

Rabbit anti-human GP73 polyclonal antibody was purchased from American Proteintech Group. Rabbit anti-human E-cadherin polyclonal antibodies and mouse anti-human Vimentin monoclonal antibody were purchased from Booster Company. Immunohistochemistry kit antibodies were purchased from Zhongshan Jinqiao Biotechnology Development Corporation.

### Major instruments

Microscope, microwave oven and medical clean bench were offered by the First Affiliated Hospital of XJMU.

### Immunohistochemistry (IHC)

Immunohistochemistry was performed using En-Vision method for IHC staining. We fixed the specimens in 10% neutral formalin and used paraffin to embed them. We obtained deparaffinized 5-μm thicksections from the FFPE tissue blocks with xylene, followed by rehydration using a graduated series of ethanol. We used 3% H_2_O_2_ to block endogenous peroxides. Microwave oven was used to block non-specific antibody binding. The sections were incubated separately overnight at 4°C with the primary antibodies, at a concentration of 1:50. The secondary antibodies were then added to the specimens the next day. DAB was used as the chromogen. The staining was terminated with water and the specimens counterstained with haematoxylin.

### Evaluation of IHC staining results

IHC staining results were interpreted independently by two pathologists blinded to the outcomes. An intensity score was assigned, representing the average intensity of positive cells (0, none; 1, weak; 2, intermediate; 3, strong). A proportion score was assigned, which represented the estimated proportion of positive-staining cells (0, no positive cells; 1, 0-20%; 2, 21-50%; 3, 51–80%; 4, 81–100%). The proportion and intensity scores were multiplied with a immunoreactivity score (IS). The IS was further divided as follows: 0–1 (−) ; 2–4 (+) ; 5–7 (++); >8(+++). “-~+” was regard as low level,“++ ~ +++” was defined as high level.

### Statistics

The appropriate non-parametric tests were used to investigate GP73 expression and clinicpathological parameters. Unvaried survival analysis was performed by the Kaplan–Meier method, and differences between the groups were analyzed by the log-rank test. Two-tailed p-values of <0.05 were considered significant. All statistical analyses were performed using SPSS software version 17.0 (SPSS for Windows, Chicago, IL, USA).

## Results

### The expression of GP73 and EMT molecules in HCC and PCL tissues and the inter-relationships

**Figure 1:**
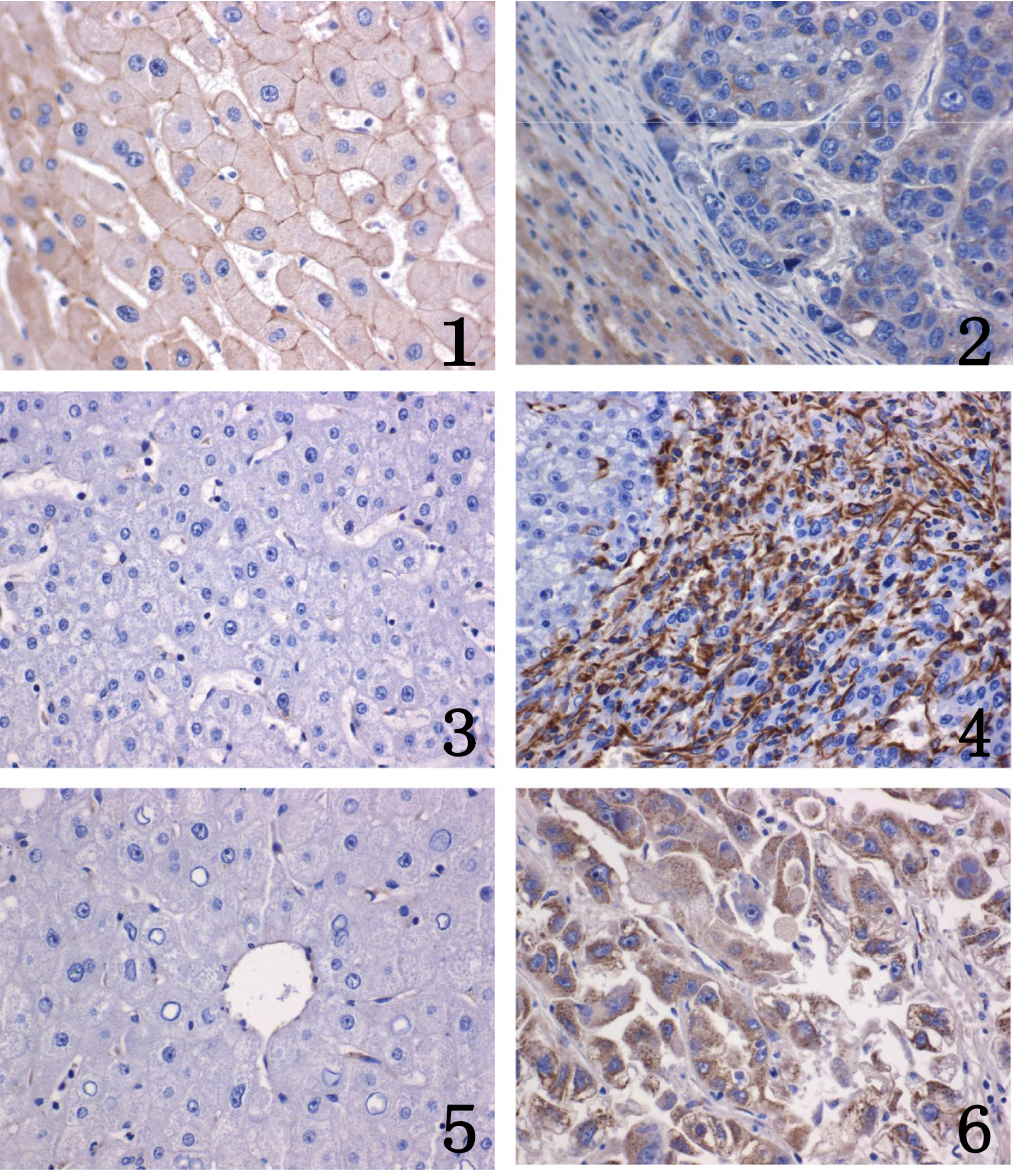
**Immunohistochemistry of E-cadherin, Vimentin and GP73 in PCL tissues and HCC tissue (400×).** E-cadherin was detected in the cell membrane. Note that E-cadherin showed moderate and strong staining in s (PCL) **(1)** By contrast, it was almost absent in normal control HCC**(2)** (×400).Vimentin was detected in the cytoplasm and interstitial cells. Note that Vimentin was almost absent in normal controls (PCL) **(3)** By contrast, moderate and strong staining was seen in HCC **(4)** (×400).GP73 protein expression was evaluated by an En-Vision immunohistochemical method. Note that GP73 was almost absent in normal controls (PCL) **(5)**. By contrast, moderate and strong staining of GP73 was identified in HCC, respectively. (×400). **(6)**.

GP73 was detected in the cytoplasm of liver cells, which is brown. E-cadherin was detected as a membrane-linear/disarrayed immunostaining pattern, while Vimentin labeling was observed in cytoplasmic and interstitial cells (Figure 1). Data showed that GP73 staining was detected up to 72% (54/75) in HCC, but only up to 4% (3/75) in the PCL tissues. GP73 expressed in HCC was higher compared with PCL (*χ*^*2*^ = 73.60, *P* < 0.05). The expression of E-cadherin in HCC was 24% (18/75) compared with PCL tissues 88% (66/75). The increased expression of Vimentin in HCC [60% (45/75)] was higher than in PCL tissues [12% (9/75)] (Table 1).

**Table 1:** GP73, E-cadherin and Vimentin in HCC and the PCL tissues

**Expression**	**N**	**Positive**	**Negative**	**Positive rate**	***χ***^***2***^	***P***
GP73						
HCC	75	54	21	72.00%		
PCL	75	3	72	4.00%	73.60	<0.05
E-cadherin						
HCC	75	18	57	24.00%		
PCL	75	66	9	88.00%	62.34	<0.05
Vimentin						
HCC	75	45	30	60.00%		
PCL	75	9	66	12.00%	37.50	<0.05

### Correlation between GP73 and clinical pathology

GP73 was associated with Edmondson grade, vascular invasion and TNM stage. ( *P* < 0.05). No correlation was detected between GP73 with gender, age, HBV infection, AFP, or thrombosis (*P* > 0.05) (Table 2).

**Table 2:** The correlation between GP73 and clinic pathologic features

**Variable**	**N**	**Positive**	**Negative**	**Positive rate**	**χ**^**2**^	***P***
Gender						
Male	51	39	12	76.47%		
Female	24	15	9	62.50%	1.58	0.21
Age						
> 60y	33	27	6	81.82%		
≤ 60y	42	27	15	64.29%	2.82	0.09
HBsAg						
Positive	27	6	21	77.78%		
Negative	48	15	33	68.75%	0.70	0.40
AFP value						
> 400 ng/ml	12	9	3	75.00%		
≤ 400 ng/ml	63	45	18	71.43%	0.06	0.80
Thrombosis						
Yes	30	21	9	70.00%		
No	45	33	12	73.33%	0.10	0.75
Edmondson grade						
I-II	15	3	12	20.00%		
III-IV	60	51	9	85.00%	25.15	<0.05
Vascular invasion						
Yes	36	18	18	50.00%		
No	39	36	3	92.31%	16.62	<0.05
TNM stage						
T 1/2	21	9	12	42.86%		
T 3/4	54	45	9	83.33%	12.29	<0.05

### GP73 expression with survival in HCC patients

**Figure 2:**
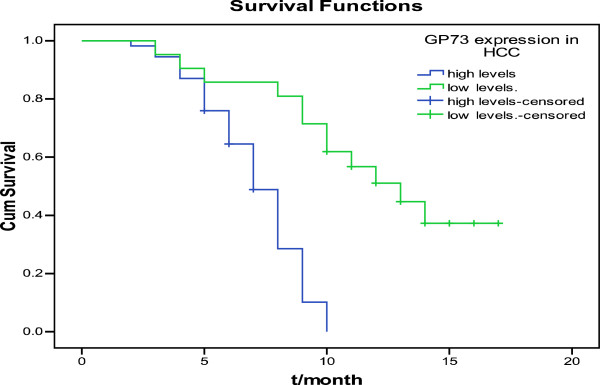
**Survival curves of HCC patients with different levels of GP73 expression.** The surival curves showed that HCC patients with high levels of GP73 expression were different from patients with low levels.

We divided the 75 specimens into high and low levels. Kaplan–Meier survival analysis and Log-rank test showed that GP73 expression was significantly associated with the OS (Figure 2, Table 3). The median survival time of patients with high levels of GP73 was 7 months compared with 13 months associated with low GP73.

**Table 3:** Kaplan–Meier survival analysis and log-rank test of GP73

**GP73**	**N**	**Median OS/month**	**95% CI**	***χ***^***2***^	***P***
High levels	54	7.00	6.25~7.75	28.18	<0.05
Low levels	21	13.00	9.27~16.73		

## Discussion

HCC is the most common primary liver cancer [9]. It is the third leading cause of cancer-related death preceded only by lung and stomach cancers [10]. Although patients with early diagnosis have benefited from advances in treatment, the prognosis in HCC is still disappointing [11], largely due to the highly invasive and metastatic potential [12]. Therefore, it is essential to find an effective biomarker for HCC prognosis and underlying mechanisms of invasion and metastasis.

GP73 is a 73 kDa type-II Golgi transmembrane glycoprotein, which was originally cloned from a library derived from patients with adult giant-cell hepatitis [2]. Research suggested that GP73 was overexpressed in HCC [3]. Mao et al reported in a large sample of international groups, that the sensitivity and specificity of GP73 in HCC were higher than AFP. It suggested that GP73 was a new type of molecular marker in early diagnosis of HCC [4]. Our data showed that tissue GP73 levels were higher level in HCC compared with PCL tissues, consistent with the preliminary studies.

In 1982, Garry Greenburg and Hay first proposed EMT [13]. Accumulating evidence indicated that EMT was a pivotal mechanism contributing to cancer invasion and metastasis. EMT involves multiple components, such as E-cadherin and Vimentin.

E-cadherin expression is a hallmark of EMT [14]. E-cadherin expression is associated with the establishment of cell polarity and tissue organization. Reduced E-cadherin expression contributes to the transition of adenoma to carcinoma in animal models and is inversely correlated with tumor stage [15-17]. Vimentin is a component of type III intermediate filaments and the archetypal mesenchymal marker most commonly used to categorize EMT. Vimentin expression is a late event in EMT, preceded by a loss of epithelial features and leading to upregulation of mesenchymal genes [18]. Huang found that Vimentin expression was significantly higher in the metastasis group than non-metastasis group ( *P* ≤ 0.05) unlike E-cadherin [19].

In the present study, IHC analysis of the expression of E-cadherin, Vimentin and GP73 showed that GP73 and Vimentin levels were higher in HCC compared with PCL tissues, unlike E-cadherin. Spearman’s correlation test indicated that GP73 was positively associated with Vimentin, but negatively with E-cadherin. The results resembled the findings of Huang et al.

Several studies have demonstrated that GP73 overexpression may be associated with tumor invasiveness [1,4,5,7,20]. Sun reported that the level of GP73 was strongly associated with tumor size, vein invasion, and tumor differentiation, which suggested that GP73 augmented tumor invasion and metastasis [20]. Our research found that GP73 was significantly correlated with the Edmondson grade, vascular invasion and TNM stage (all *P* < 0.05) (Table 2). These clinicopathological features are characteristic of tumor invasiveness and metastatic potential. In conjunction with the relationships among GP73 and EMT, we hypothesize that GP73 might be associated with invasive behavior in HCC.

In this study, survival analysis showed that patients with high level of GP73 showed poorer OS compared with low levels. We presumed that GP73 might correlate with patients’ prognosis, contrary to Sun et al. Notably, Sun proved that serum GP73 has no correlation with prognosis, but the correlation of tissue GP73 and prognosis are unknown. There exist a differentiation between serum and tissue GP73, more studies are needed to prove the prognostic value of GP73. And the results may be affected by the number of specimens, histological type and pathological grading method [21], different results were permitted.

Our study limitations relate to the small sample size of the patients’ cohort with only 75 patients. The group size became even smaller when divided by the high level and low level of GP73 expression. Nonetheless, multiple logistic regression analysis was applied to assess parameters independently associated with GP73.

## Conclusions

In summary, GP73 expression positively correlated with EMT molecules. High level of GP73 was associated with Edmondson grade, vascular invasion and TNM stage. Therefore, we conclude that GP73 involved in the regulation of EMT to obtain invasion on HCC and it would be a biomarker for assessing the prognostic values. GP73 is a prognostic biomarker. However, The regulation of GP73 and its function are controversial and have yet to be clarified [22]. How its occurrence in the HCC still needs to be elucidated.

## Competing interests

We certify that we have participated sufficiently in the work to take public responsibility for the appropriateness of the experimental design and method, and the collection, analysis, and interpretation of the data. We have reviewed the final version of the manuscript and approve it for publication. To the best of our knowledge and belief, this manuscript has not been published in whole or in part nor is it being considered for publication elsewhere.

## Authors’ contributions

Y-XB, QC and YY have made substantial contributions to conception and design, and acquisition and analysis of data. RM and LX have been involved in drafting the manuscript or revising it critically for important intellectual content. Y-XB has given final approval of the version to be published. All authors read and approved the final manuscript.
